# Estimation of optimal ratios of digestible phenylalanine + tyrosine, histidine, and leucine to digestible lysine for performance and breast yield in broilers

**DOI:** 10.3382/ps/pew305

**Published:** 2016-09-19

**Authors:** Sebastian M. Franco, Fernando de C. Tavernari, Rosana C. Maia, Victor R. S. M. Barros, Luiz F. T. Albino, Horacio S. Rostagno, Guilherme R. Lelis, Arele A. Calderano, Ryan Neil Dilger

**Affiliations:** * Premex S.A. Medellin, Anquioquia, Colombia; † EMBRAPA Swine and Poultry, Concordia, SC, Brazil; ‡ Department of Animal Science, Federal University of Viçosa, Viçosa, MG, Brazil; § Department of Animal Sciences and Division of Nutritional Sciences, University of Illinois, Urbana, Illinois

**Keywords:** digestible amino acid, broiler, phenylalanine + tyrosine, histidine, leucine

## Abstract

Three experiments were carried out to estimate the optimal ratios of digestible phenylalanine + tyrosine (Phe + Tyr), histidine (His), and leucine (Leu) relative to digestible lysine (Lys) for performance and carcass criteria of Cobb-500 broilers from 8 to 17 d of age. In each experiment, 160 male chicks were allocated to a completely randomized experimental design with eight replicate pens, each receiving five dietary treatments. A common, semi-purified basal diet was formulated to meet all dietary recommendations except for those of the tested amino acids (i.e., Phe + Tyr, His, and Leu). Growth performance and carcass characteristics data were analyzed using various requirement-estimation models, including 95% of the quadratic regression, linear response plateau (LRP; i.e., stepwise regression), LRP-to-quadratic regression ratio; and quadratic broken line (QBL). Graded digestible Phe + Tyr ratios elicited a quadratic response (*P* < 0.05) in body weight gain and linear responses (*P* < 0.05) in breast and breast fillet weights. Linear effects (*P* < 0.05) were also observed when graded ratios of digestible His were fed for feed intake and weight gain, and quadratic responses (*P* < 0.05) were noted for feed conversion ratio and breast and breast fillet weights and yields. Graded Leu ratios elicited quadratic responses (*P* < 0.05) in feed intake, weight gain, and breast and breast fillet weight and yield. Based on growth and carcass parameters, the estimated ideal digestible ratios of Phe + Tyr, His, and Leu relative to digestible Lys were 112, 38, and 104%, respectively, for broiler chicks raised from 8 to 17 d of age.

## INTRODUCTION

Increasing economic pressures in poultry production have focused attention on reducing the production costs, in which dietary inputs represent the major portion. Protein remains the most expensive dietary nutrient, though the use of crystalline amino acids offers multiple advantages in that they provide reductions in both dietary crude protein concentrations and the excretion of dietary nitrogen into the environment. Currently, most broiler diets are formulated using the ideal protein concept, in which all the amino acids are fed at optimal ratios relative to the dietary lysine (**Lys**) concentration. However, insufficient estimates for optimal ratios currently exist for phenylalanine + tyrosine (**Phe + Tyr**), histidine (**His**), and leucine (**Leu**) when included in starter-broiler diets.

The typical order of first limiting amino acids for maximal protein synthesis in corn-soybean meal-based diets for broilers includes methionine, lysine, threonine, and valine, and, for this reason, extensive research efforts have been put forward on these amino acids (Schwartz and Bay, 1975; Han et al., 1992; Fernandez et al., 1994; Corzo et al., 2008). However, little is known about the optimal dietary standards of Leu, His, and Phe, although they also must be provided in adequate amounts to optimize broiler growth (Wecke and Liebert, 2013). As a branched-chain amino acid, Leu has an important role in the metabolism of others amino acids. The excess of Leu in low-protein diets increases the catabolism of valine (Val) and isoleucine (Ile) leading to a deficiency of these amino acids for adequate growth. Moreover, Leu has been proven to uniquely exert positive effects on lean tissue accretion through increased initiation of protein synthetic pathways (Anthony et al., 2000; Rajendram et al., 2015).

His is an integral component of a broad set of tissues including skin, feather, bone, ligaments, and, obviously, muscle (NRC, 1994). This amino acid also serves to stimulate the digestive secretion of gastrin, a hormone that activates production of hydrochloric acid and pepsinogen, which are essential for digestion of dietary protein (Berdanier, 1998; D’Mello, 2003). Phe is important for the synthesis of thyroid hormones that control metabolic processes, thereby influencing the growth of different body structures; feed efficiency; oxygen consumption; synthesis and metabolism of proteins, carbohydrates and lipids; thermogenesis; and acclimation to environmental changes (Gropper and Smith, 2012).

The optimal ratios of Leu, His, and Phe + Tyr with digestible Lys respectively for starter phase in the literature are 107%, 27%, 119% (Dean and Scott, 1965), 126%, 32%, 95% (Huston and Scott, 1968), 110%, 36%, 100% (Sasse and Baker, 1973), 109%, 36%, 100% (Baker and Han, 1994), and 107%, 36%, and 115% (Dorigam et al., 2013).

Although functional requirements for these amino acids clearly seem to maximize growth performance and carcass characteristics of fast-growing broilers, there remains a lack of quantifiable information as to optimal dietary ratios. Thus, the objective of this study was to determine the optimal ratios of digestible Phe + Tyr, His, and Leu relative to digestible Lys in male broiler chicks during the starter period, from 8 to 17 d of age, with primary response outcomes including live performance and breast yield.

## MATERIALS AND METHODS

All experimental procedures were previously approved by the Ethics Committee on Animal Use (protocol no. 32/2010), which is consistent with the ethical principles of animal experimentation established by the Brazilian College of Animal Experimentation (COBEA, 1991).

In total, three experiments were carried out on the Poultry Farm of the Federal University of Viçosa. In each experiment, 160 male Cobb-500 broiler chicks were used during the starter phase (8 to 17 d of age). From 1 to 7 d post-hatch, birds were housed in a masonry house divided into pens (1.0 m × 2.0 m) with wood-shavings litter and provided with nipple drinkers and bucket feeders with ad libitum access to feed and water. Broilers were fed a pre-starter diet based on corn and soybean meal formulated to meet the nutrient recommendations proposed by Rostagno et al. (2005), and birds were managed according to the breeder recommendations. On d 8 post-hatch, birds were weighed and randomly allotted to dietary treatments such that the average initial pen weights and weight distributions were similar across treatments. Five dietary treatments were fed to eight replicate pens of four birds in each experiment, each including five graded digestible amino acid concentrations to produce specific ratios relative to the digestible Lys concentration of the basal diet. The following digestible ratios were tested: Phe + Tyr (94, 100, 106, 112, or 118%; experiment 1), His (28, 31, 34, 37, or 40%; experiment 2), and Leu (93, 100, 107, 114, or 121%; experiment 3).

A common, semi-purified basal diet (Table 1) was formulated using corn starch, broken rice, fish meal, and soybean meal to meet nutritional recommendations (Rostagno et al., 2005), except for Phe + Tyr, His, or Leu, in experiments 1, 2, and 3, respectively. The broken rice, fish meal, soybean meal, and meat and bone meal amino acids were analyzed prior to mixing the common basal or final dietary treatments. The feedstuffs used in the basal diet were analyzed for crude protein and total amino acid, and the digestible amino acids were calculated using the standardized ileal digestibility (**SID**) coefficient with broilers in starter phase (Rostagno et al., 2005). The crystalline amino acids were considered to have 100% SID. Upon conclusion of the study, chicks and feeders were weighed, and body weight gain (**WG**), feed intake (**FI**), and feed conversion ratio (**FCR**) were calculated for each replicate pen of chicks. Broilers were sacrificed by cervical dislocation, and carcasses were evaluated for breast (**BrW**) and breast fillet (i.e., deboned breast) weights, which were also expressed relative to carcass weight (i.e., yield).

**Table 1.** Ingredients and calculated nutrient composition of the basal diet.

Ingredient	Amount (g/kg)
Corn, 7.85%	20.0
Broken rice	180.0
Soybean meal, 45%	150.0
Fish meal, 45%	70.0
Meat and bone meal, 51%	50.0
Glutamic acid	70.0
Corn starch	359.0
Washed sand	30.0
Soybean oil	30.0
Potassium carbonate	4.0
Sodium bicarbonate	4.0
Salt	2.44
L-lysine HCl, 99%	4.11
DL-methionine, 99%	4.15
L-threonine, 98%	2.40
L-arginine, 98.5%	2.20
L-valine, 99%	2.10
L-isoleucine, 98.5%	1.80
L-phenylalanine, 99%	1.37 (0.00)^1^
L-histidine HCl, 74%	1.14 (0.00)^2^
L-leucine, 99%	1.61 (0.00)^3^
L-tyrosine, 99%	0.91
L-glycine	4.00
L-tryptophan, 98%	0.46
Premix^4^	4.05
*Analyzed composition*	
Crude protein, g/kg	200.9
Metabolizable energy, kcal/kg	3,188
Calcium, g/kg	9.10
Available phosphorus, g/kg	4.70
Digestible amino acids, g/kg	
Lys	10.50 (10.31)^5^
Met + Cys	7.69
Met	6.16
Thr	7.03
Trp	2.30
Arg	11.50
Val	8.18
Ile	7.15
Phe + Tyr	10.05 (9.46)^5^
His	3.00 (2.74)^5^
Leu	9.95 (9.96)^5^

^1^Basal diet of the experiment on digestible Phe + Tyr-to-Lys ratio.

^2^Basal diet of the experiment on digestible His-to-Lys ratio.

^3^Basal diet of the experiment on digestible Leu-to-Lys ratio.

^4^Mineral premix (amount per kg diet): manganese - 77.0 mg, iron - 55.0 mg, zinc - 71.5 mg, copper - 11.0 mg, iodine - 1.10 mg, and excipient q.s. - 1,000 g; Vitamin premix (amount per kg diet): vitamin A - 8250 IU, vitamin D3 - 2090 IU, vitamin E - 31.0 IU, vitamin B1 - 2.20 mg, vitamin B6 - 3.08 mg, pantothenic acid - 11.0 mg, biotin - 0.077 mg, vitamin K3 - 1.65 mg, folic acid - 0.77 mg, nicotinic acid - 33.0 mg, vitamin B12 - 0.013 mg, selenium - 0.33 mg, and excipient q.s. - 1,000 g); 1 g choline chloride 60%/kg diet; 0.1 g butylated hydroxytoluene/kg diet; 0.55 g sodium salinomycin 12%/kg diet; 0.1 g avilamycin 10%/kg diet.

^5^Values in parentheses indicate analyzed dietary concentration of the amino acid studied in the respective experiments.

### Statistical Analysis

All data were initially subjected to a 1-way ANOVA using the PROC MIXED procedure of SAS/STAT software (version 9.2; SAS Institute, 2009). Subsequently, each response parameter was modeled using only linear and quadratic regression analyses, as no higher-order polynomials were observed to be significant with alpha = 0.05. When quadratic effects were observed, an optimal ratio was estimated at 95% of the maximum or minimum response to avoid overestimation (Sakomura and Rostagno, 2007).

Additionally, a linear response plateau (**LRP**) model was used to determine the optimal ratios for each evaluated parameter (Pesti et al., 2009). Finally, a quadratic broken-line (**QBL**) regression was computed for each response using the NLIN procedure (Robbins et al., 2006). When the same dependent variable exhibited both LRP and quadratic responses, the ratio of these two optimal estimates was taken to define another estimated optimal ratio. Finally, overall-mean optimal digestible ratios of Phe + Tyr, His, and Leu were estimated among the following models: linear regression (linear or quadratic effects), 95% of the quadratic response, LRP, LRP/95% of quadratic response ratio, and QBL.

## RESULTS

### Experiment 1: Phe + Tyr

No effects of digestible Phe + Tyr ratios were observed on feed intake, feed conversion ratio, breast yield (**BY**), or breast fillet yield (**BFY**) of broilers during this experiment. However, there was a linear effect (*P* < 0.05) on WG, BrW, and breast fillet weight (**BFW**), as shown in Table 2.

**Table 2.** Effect of digestible Phe + Tyr ratios on growth performance and carcass characteristics of broilers during the starter phase.^1^

	Digestible Phe + Tyr ratio, %^2^						

Variable	94	100	106	112	118	SEM	*P*-value
Feed intake, g	490.1	496.8	503.1	507.6	502.9	3.183	0.16
Weight gain, g^*^	359.6	378.4	385.4	391.4	389.3	2.153	0.023
Feed conversion ratio, g/g	1.318	1.315	1.306	1.296	1.290	0.004	0.15
Breast weight, g^**^	103.5	107.6	107.9	109.4	109.9	0.603	0.021
Breast fillet weight, g^**^	80.6	82.5	83.4	84.8	85.4	0.541	0.042
Breast yield, %	18.77	18.90	19.00	19.04	19.36	0.059	0.60
Breast fillet yield, %	14.58	14.70	14.71	14.77	15.06	0.058	0.72

^*^Linear response to graded digestible Phe + Tyr ratio (*P* < 0.05).

^**^Quadratic response to graded digestible Phe + Tyr ratio (*P* < 0.05).

^1^Values are means of 8 replicate pens of 4 male chicks from 8 to 17 d of age.

^2^All birds received a common pre-starter diet from day 1 to 7 post-hatch, and all diets (pre-starter and starter) met or exceeded nutrient recommendations for each age of chicks (Rostagno et al., 2005). In this experiment, crystalline Phe + Tyr was added replacing corn starch to produce the desired digestible concentrations of these amino acids relative to digestible Lys.

**Figure 1.**
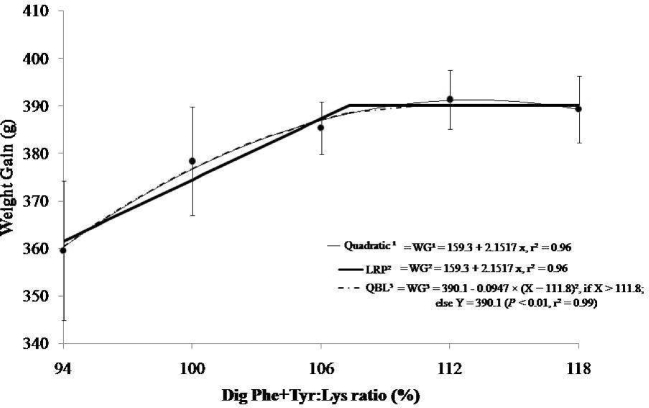
Modeled effects of dietary digestible Phe + Tyr:Lys ratios (%) on body weight gain of broiler chicks in the feeding period from 8 to 17 d of age. The digestible Phe + Tyr-to-digestible Lys ratios by quadratic regression on body weight gain (y = –695.14 + 19.21x – 0.0849x^2^, r^2^ = 0.99) produce a maximal digestible ratio estimate of 113%, or 107% at 95% of this response. Using the LRP model, an optimal ratio of 107% was estimated, with a plateau WG response of 390.4 g and an increasing slope to that plateau (y = 159.3 – 2.1517x, r^2^ = 0.96). The QBL model was also significant (*P* < 0.01) for body WG with a plateau response of 390.1 g [y = 390.1 – 0.0947 × (x − 111.8)^2^, when x > 111.8; otherwise, 390.1; r^2^ = 0.99].

For body weight gain, all models, except for the simple linear model, were significant (*P* < 0.05; Figure 1). Regarding the dietary digestible Lys concentration, graded digestible Phe + Tyr elicited a quadratic response in body weight gain (y = –695.14 + 19.21x – 0.0849x^2^, r^2^ = 0.99) to produce a maximal digestible ratio estimate of 113%, or 107% at 95% of this response. Using the LRP model, an optimal ratio of 107% was estimated, with a plateau WG response of 390.4 g and an increasing slope to that plateau (y = 159.3 − 2.1517x, r^2^ = 0.96). Expressed in relation to each other, the LRP and 95% of the quadratic responses resulted in an optimal digestible Phe + Tyr ratio of 110%. Finally, the QBL model was also significant (*P* < 0.01) for body WG with a plateau response of 390.1 g (y = 390.1 – 0.0947 × (x − 111.8)^2^, when x > 111.8; r^2^ = 0.99).

### Experiment 2: His

Feeding broilers graded digestible His ratios elicited a linear responses (*P* < 0.05) in FI and WG, and quadratic responses in FCR, as well as breast and breast fillet weights and yields (Table 3). A significant effect (*P* < 0.05) of the different evaluated digestible His-to-Lys ratios was noted on feed intake, which increased linearly, as determined by the equation FI = 307.61 + 5.1133x (r^2^ = 0.78). Weight gain had a linear influence (*P* < 0.05), by the equation WG = 218.98 + 4.25x, r^2^ = 0.79, which determined a digestible His-to-Lys ratio of 40% for maximum weight gain.

**Table 3.** Effect of digestible histidine ratios on growth performance and carcass characteristics of broilers during the starter phase.^1^

	Digestible histidine ratio, %^2^						

Variable	28	31	34	37	40	SEM	*P*-value
Feed intake, g^*^	436.0	476.7	491.1	504.9	498.6	3.361	0.002
Weight gain, g^*^	328.1	353.2	376.6	382.1	377.4	2.628	0.001
Feed conversion ratio, g/g^**^	1.371	1.317	1.305	1.323	1.321	0.003	0.027
Breast weight, g^**^	81.9	93.1	100.5	107.6	106.8	0.545	0.006
Breast fillet weight, g^**^	58.2	69.0	75.4	82.8	82.7	0.485	0.001
Breast yield, %^**^	16.05	17.35	18.21	19.30	19.31	0.056	0.014
Breast fillet yield, %^**^	11.40	12.87	13.65	14.82	14.94	0.049	0.005

^*^Linear response to graded digestible His ratio (*P* < 0.05).

^**^Quadratic response to graded digestible His ratio (*P* < 0.05).

^1^Values are means of 8 replicate pens of 4 male chicks from 8 to 17 d of age.

^2^All birds received a common pre-starter diet from day 1 to 7 post-hatch, and all diets (pre-starter and starter) met or exceeded nutrient recommendations for each age of chicks (Rostagno et al., 2005). In this experiment, crystalline histidine was added replacing corn starch to produce the desired digestible concentrations of these amino acids relative to digestible Lys.

Graded digestible His ratios elicited a quadratic response (*P* < 0.05) in feed conversion ratio (y = 2.5251 – 0.0681x + 0.001x^2^, r^2^ = 0.88), which provided an estimated optimal ratio of 36%, or 34% when taken at 95% of the quadratic asymptote. Based on the LRP model (y = 1.3175 + 0.0215, r^2^ = 0.97), the optimal digestible His ratio for FCR was estimated at 31%, and the ratio of the LRP and 95% of the quadratic models provided an optimal estimate of 34%. The QBL model (y = 1.3167 – 0.00333 × (x − 32)^2^, when x > 32; otherwise, FCR = 1.3167 (*P* < 0.03, r^2^ = 0.97) showed 32% as the best ratio.

The average digestible His-to-Lys ratio calculated from the different statistical models in the present study (33%) for feed conversion ratio was higher than the 32% obtained by Baker and Han (1994), who worked with broilers between 8 and 22 d of age.

**Figure 2.**
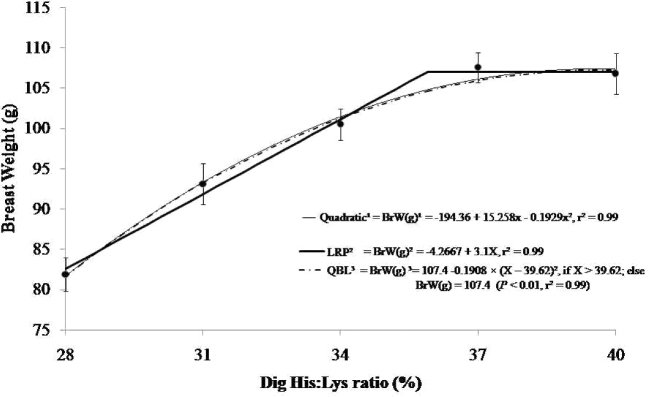
Modeled effects of dietary digestible His:Lys ratios (%) on breast weight of broiler chicks in the feeding period from 8 to 17 d of age. The digestible His-to-Lys ratios by quadratic regression on breast weight (y = –194.36 + 15.258x − 0.1929x^2^, r^2^ = 0.99) produce a maximal digestible ratio estimate of 39%, or 37% at 95% of this response. Using the LRP model, an optimal ratio of 36% was estimated, with a plateau BrW response of 107.3 g and an increasing slope to that plateau (y = –4.2667 + 3.1X, r^2^ = 0.99). The QBL model was also significant (*P* < 0.01) for body WG, with a plateau response of 107.4 g [y = 107.4 – 0.1908 × (X – 39.62)^2^, if X > 39.62; otherwise, BrW (g) = 107.4 (*P* < 0.01; r^2^ = 0.99].

The applied digestible His-to-Lys ratios had a quadratic effect (*P* < 0.05) on BrW and BFW, as shown by the equation BrW (g) = –194.36 + 15.258x – 0.1929x^2^ (r^2^ = 0.99) and BFW (g) = –185.41 + 13.319x – 0.1651x^2^ (r^2^ = 0.99). The optimal ratios found were 39% and 41%, respectively, but when a 95% coefficient was applied to the quadratic equation, the estimated requirement was 37% for breast weight and 39% for breast fillet. The QBL regression was BrW (g) = 107.4 – 0.1908 × (X – 39.62)^2^, if X > 39.62; otherwise, BrW (g) = 107.4 (*P* < 0.01, r^2^ = 0.99). According to the above equation, the digestible histidine to lysine ratio was 40%. Applying QBL on BFW, a digestible His-to-Lys ratio of 40% was found (BFW (g) = 83.23 – 0.1651 × (X – 40)^2^, if X > 40.3; otherwise, BFW (g) = 83.23 (*P* < 0.01, r^2^ = 0.99). The best digestible His-to-Lys ratio determined for the LRP model was 36% with a plateau at 107.3 g for breast weight (y = –4.2667 + 3.1X) and the same ratio for breast fillet (y = –21.3344 + 2.8667X). When the results of the quadratic equation and the LRP model were compared, the best digestible histidine to lysine ratios for breast weight and breast fillet were 38% and 39%, respectively (Figure 2).

The BY and BFY of broilers during the starter phase had a quadratic effect (*P* < 0.05) with different levels of digestible His-to-Lys ratio, as shown by the equations BY (%) = –12.78 + 1.5506x – 0.0187x^2^ (R^2^ = 0.99) and BFY (%) = –17.561 + 1.5477x – 0.0183x^2^ (R^2^ = 0.99), which determined 41% and 39% for maximum breast yield and breast fillet yield, respectively. Applying 95% coefficient of quadratic equation response, an optimal ratio of 39% was determined for breast yield and 40% digestible His-to-Lys ratio for breast fillet yield.

In the QBL adjustment, the digestible His-to-Lys ratios for BY and BFY of chicks were 41% and 42%, respectively, as shown by the equations BY(%) = 19.45 – 0.0187 × (X – 41.50)^2^, if X > 41.5; otherwise, BY (g) = 19.45 (*P* < 0.01, r^2^ = 0.99) and BFY(%) = 15.10 – 0.0183 × (X – 42.21)^2^, if X > 42.21; otherwise, BY (g) = 15.10 (*P* < 0.01, r^2^ = 0.99). According to the LRP analysis, the best digestible His-to-Lys ratio for breast yield and breast fillet yield was 37%.

### Experiment 3: Leu

There was no significant effect (*P* > 0.05) of the evaluated digestible Leu-to-Lys ratios on feed conversion ratio, but feed intake, weight gain, feed conversion ratio as well as carcass parameters (breast and breast fillet weight and yield) had a quadratic response, as shown in Table 4.

**Table 4.** Effect of digestible leucine ratios on growth performance and carcass characteristics of broilers during the starter phase.^1^

	Digestible leucine ratio, %^2^						

Variable	93	100	107	114	121	SEM	*P*-value
Feed intake, g^*^	428.6	489.6	492.2	499.8	498	1.823	0.001
Weight gain, g^*^	323.9	373	389.3	386.7	376.5	1.436	0.001
Feed conversion ratio, g/g	1.332	1.313	1.307	1.297	1.323	0.003	0.51
Breast weight, g^*^	88.8	112.4	115.7	113.9	109.7	0.408	0.001
Breast fillet weight, g^*^	67.2	85.2	89.4	85.7	82.3	0.401	0.001
Breast yield, %^*^	17.26	19.68	20.01	19.56	19.26	0.060	0.001
Breast fillet yield, %^*^	12.99	14.91	15.46	14.69	14.46	0.060	0.001

^*^Quadratic response to graded digestible Leu ratio (*P* < 0.05).

^1^Values are means of 8 replicate pens of 4 male chicks from 8 to 17 d of age.

^2^All birds received a common pre-starter diet from day 1 to 7 post-hatch, and all diets (pre-starter and starter) met or exceeded nutrient recommendations for each age of chicks (Rostagno et al., 2005). In this experiment, crystalline leucine was added replacing corn starch to produce the desired digestible concentrations of these amino acids relative to digestible Lys.

**Table 5.** Summary of estimated optimal digestible amino acid ratios for broiler chicks.

		Model^1^						Mean

Amino acid	Response	L	Q	95% Quad	LRP	LRP/Quad	QBL	ratio^2^
Phe + Tyr	Body weight gain	–	113	107	107	110	112	
	Breast weight	118	–	–	–	–	–	112
	Breast fillet weight	118	–	–	–	–	–	
His	Body weight gain	40	–	–	–	–	–	
	Feed conversion ratio	–	36	34	31	34	32	
	Breast weight	–	39	37	36	38	40	38
	Breast fillet weight	–	41	39	36	39	40	
	Breast yield	–	41	39	37	39	41	
	Breast fillet yield	–	39	40	37	38	42	
Leu	Body weight gain	–	111	106	102	106	105	
	Breast weight	–	110	105	99	104	101	
	Breast fillet weight	–	110	105	99	104	101	104
	Breast yield	–	110	105	99	105	100	
	Breast fillet yield	–	109	104	99	105	100	

^1^Optimal digestible ratio estimates shown for each response in which at least one model was significant. Abbreviations: L, linear; Q, quadratic; 95% Quad, 95% of the asymptotic parameter of the quadratic model; LRP, linear response plateau; LRP/Quad, LRP-to-quadratic regression ratio; QBL, quadratic broken line.

^2^Overall-mean estimated optimal digestible ratio across all significant response variables per amino acid.

The applied digestible Leu-to-Lys ratios had a quadratic effect on feed intake, as shown by the equation FI = –1741.6 + 39.75x – 0.1758x^2^, r^2^ = 0.91, with a higher ratio of 113%. Applying the 95% coefficient of quadratic equation response, a higher ratio, of 107%, was determined. When the LRP model was applied, the best digestible Leu-to-Lys ratio determined was 99%, with a plateau at 495 g (y = –599.05 + 11.05). When the results of the quadratic equation and the LRP model were compared, the best digestible Leu-to-Lys ratio for feed intake was 106%. In adjusting the QBL model, the best digestible Leu-to-Lys ratio was 103%, given by the equation FI (g) = 496.7 – 0.6382 × (X – 103.3)^2^, if X > 103.3; otherwise, FI (g) = 496.7 (*P* < 0.01, r^2^ = 0.99).

**Figure 3.**
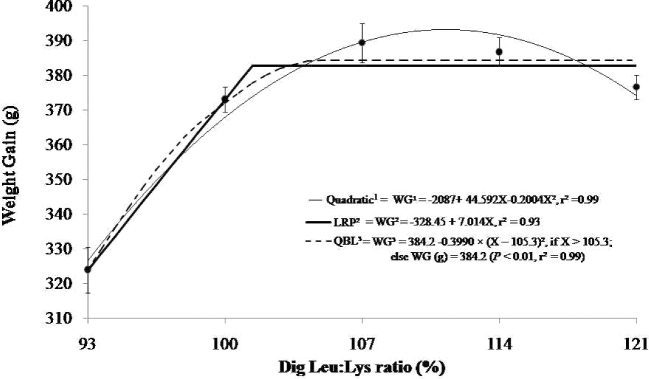
Modeled effects of dietary digestible Leu:Lys ratios (%) on body weight gain of broiler chicks in the feeding period from 8 to 17 d of age. The digestible Leu-to-digestible Lys ratios by quadratic regression on body weight gain (y = –2087 + 44.592x – 0.2004x^2^, r^2^ = 0.98) produce a maximal digestible ratio estimate of 111%, or 106% at 95% of this response. Using the LRP model, an optimal ratio of 102% was estimated, with a plateau WG response of 384.0 g and an increasing slope to that plateau (y = 328.45 + 7.014, r^2^ = 0.93). The QBL model was also significant (*P* < 0.01) for body WG with a plateau response of 384.2 g [WG (g) = 384.2 – 0.3990 × (X – 105.3)^2^, if X > 105.3; otherwise, WG (g) = 384.2 (*P* < 0.01, r^2^ = 0.99].

Applying regression analyses, the digestible Leu-to-Lys ratio had a quadratic effect (*P* < 0.05) on weight gain (y = –2087 + 44.592x – 0.2004x^2^, r^2^ = 0.98), with an optimal ratio of 111% (Figure 3), which corresponds 393.58 g. Applying the 95% confidence level to the quadratic equation response, an optimal ratio of 106% was determined. For the LRP model, the best digestible Leu-to-Lys ratio determined was 102%, with a plateau at 384 g (y = –328.45 + 7.014, r^2^ = 0.93). When the results of the quadratic equation and the LRP model were compared, the best digestible Leu-to-Lys ratio for weight gain (g) was 106%. Using the QBL model, the digestible Leu-to-Lys ratio was 105% by the equation WG (g) = 384.2 – 0.3990 × (X – 105.3)^2^, if X > 105.3; otherwise, WG (g) = 384.2 (*P* < 0.01, r^2^ = 0.99). Analyzing regression points obtained using different models on performance parameters, the values found are consistent with the 109 and 108% recommended by Baker and Johnson (1999) and Rostagno et al. (2005), respectively.

Breast weight and breast fillet weight had the same optimal Leu-to-Lys ratios of 110% for the quadratic model (BrW = –962.47 + 19.554x – 0.0885x^2^, r^2^ = 0.93; BFW = –803.88 + 16.255x – 0.0739x^2^, r^2^ = 0.93) and 99% for the LRP model (BrW = –472.13 + 6.0313X; r^2^ = 0.97; BFW = 15.72 + 0.0394x, r^2^ = 0.92). The estimated optimal average (Q/LRP) was 104%. Using the QBL model, the digestible Leu-to-Lys ratio found was 101% for BrW and BFW (BrW(g) = 113.1 – 0.3419 × (X – 101)^2^, if X > 101; otherwise, BrW (g) = 113.1 (*P* < 0.01, r^2^ = 0.99) and BFW(g) = 85.8 – 0.2555 × (X – 101.5)^2^, if X > 101.5; otherwise, BrW (g) = 85.8 (*P* < 0.01, r^2^ = 0.99)).

**Figure 4.**
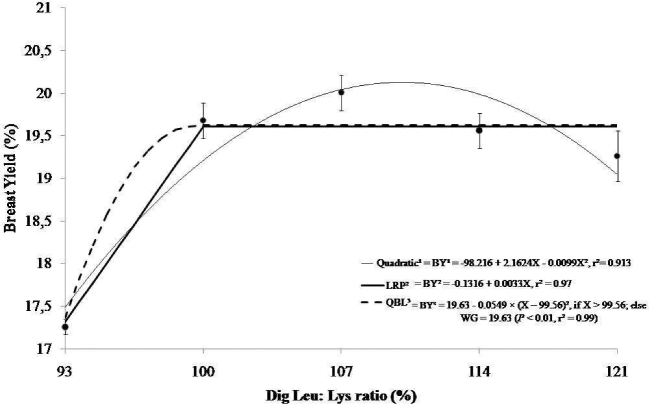
Modeled effects of dietary digestible Leu:Lys ratios (%) on breast yield of broiler chicks in the feeding period from 8 to 17 d of age. The digestible Leu to digestible Lys ratios by quadratic regression on body breast yield (y = –98.216 + 2.1624x + 0.0099x^2^, r^2^ = 0.91) produce a maximal digestible ratio estimate of 110%, or 105% at 95% of this response. Using the LRP model, an optimal ratio of 99% was estimated, with a plateau WG response of 19.51 g and an increasing slope to that plateau (y = –0.1316 + 0.0033x, r^2^ = 0.93). The QBL model was also significant (*P* < 0.01) for body BY, with a plateau response of 19.63 g [BY (g) = 19.63 – 0.0549 × (X – 99.56)^2^, if X > 99.56; otherwise, BY (g) = 19.63 (*P* < 0.01, r^2^ = 0.99].

The increased digestible Leu-to-Lys ratios affected the BY of the broilers aged 8 to 17 d. Both the quadratic (110%) and linear response plateau (99%) affected BY, which reached a maximum of 19.63 g. The LRP (99%) and the quadratic regression (110%) gave estimated optimal average Leu-to-Lys ratios for BY of 105% in 8 to 17d old broilers. Fitting the QBL, a digestible Leu-to-Lys ratio of 100% was found (Figure 4).

The improved BrW and BFW with increased WG observed at higher Leu-to-Lys ratios suggested that the ratio had a significant effect on breast yield and breast fillet yield. When analyzed by the quadratic regression (BFY = –82.648 + 1.792x –0.0082x^2^, r^2^ = 0.88) and LRP (BFY = –12.12 + 0.27X), the optimal Leu-to-Lys ratios for breast fillet yield were 109% and 100%, respectively. Applying 95% coefficient of the quadratic equation response, an optimal ratio of 104% was determined. The optimal value for BFY using QBL was 100% (BFY (g) = 14.88 – 0.388 × (X – 100)^2^, if X > 100; otherwise, BFY (g) = 14.88 (*P* < 0.01, r^2^ = 0.99)).

## DISCUSSION

Many statistical models have been developed to determine the requirements of amino acids and proteins in humans (Elango et al., 2012) and animals, but the choice of method will depend essentially on the species to be worked on, application facilities, and best fit of the data. This adjustment may produce different amino acid ratio recommendations (Lelis et al., 2014). According to Euclydes and Rostagno (2001), the choice of the model depends on the relationship between the levels of the studied nutrient and the evaluated responses. The optimal level may be underestimated, as in the case of the LRP model. However, although the quadratic function seems to have advantages when determining nutritional requirements because it estimates the maximum possible performance, it is very sensitive to the evaluated levels and has bilateral symmetry, which may be not adequate biologically. The quadratic broken line models represent theoretical ideas of the nature of nutritional responses more closely than multiple range tests or polynomial models do. The classical concept of “requirement” is clearly defined as the nutrient input level resulting in maximum response for variables like growth, or a minimum for variables like carcass. The curved ascending segment represents biological responses more realistically (Pesti et al., 2009).

The complexity in establishing precise amino acid ratios, according to Baker et al. (2002), lies in the possibility of different parameters (weight gain, breast weight, breast fillet weight, breast yield, and breast fillet yield) and statistical models being used. Those authors used the quadratic model with plateau and demonstrated that it can be used to determine amino acid ratios, because they obtained values close to 90% of those calculated using quadratic equations. Baker et al. (2002) stated that the combination of models by using the quadratic equation associated with the response plateau may yield the best recommended level.

The digestible Phe-to-Lys ratio of 112% (Table 5) determined in experiment 1 was similar to results found by NRC (1994) of 112%, higher than results found by Baker and Han (1994) of 105%, and lower than results found by Dorigam et al. (2013) of 115%. The quadratic effect observed for weight gain may be associated with the effects of excess Phe that could decrease the transport of others amino acids, especially tryptophan, from the blood-brain barrier, and may reduce serotonin levels (Peganova et al., 2003).

Excess of Phe in diets fed to chickens results in retarded growth and development of physical parameters (Tamimie and Pscheidt, 1966). In the Phe + Tyr requirement, approximately 55% are estimated to be provided by Phe (Rostagno et al., 2011). In diets adequate in Phe, but deficient in Tyr, Phe could be equal in efficacy to Tyr in providing the limiting amino acid (Sasse and Baker, 1972), Tyr. Phe deficiency decreases the activity of hepatic phenylalanine hydroxylase and consequently the levels of Tyr (Lartey and Austic, 2009).

In experiment 2, the digestible His-to-Lys was higher than the 26%, 29%, and 36% reported by Hurwitz et al. (1978), Baker and Han (1994), and Rostagno et al. (2005). However, in calculating the average between performance and carcass characteristics, the digestible His-to-Lys ratio found was lower than the 40% reported by Scott and Austic (1982) and NRC (1994) as the optimal digestible His-to-Lys ratio for broiler performance. Herwitt and Lewis (1972) found a quadratic effect of histidine supply in chicken diets on feed efficiency at the His-to-Lys ratios of 31% or 47%.

His stimulates the digestive secretion of gastrin, a hormone that activates the production of hydrochloric acid and pepsinogen (Berdanier, 1998) by the histamine production pathway, thereby influencing nutrient utilization and growth performance.

There are different recommendations in the literature as regards the digestible Leu-to-Lys ratio for starter broilers. According to Baker et al. (2002), the accuracy of amino acid ratio determinations depends on the type of parameter used, such as weight gain and feed conversion ratio, as well as on the statistical analyses applied. Hurwitz et al. (1978), Scott and Austic (1982), Baker and Han (1994), NRC (1994), and Rostagno et al. (2005) reported digestible Leu-to-Lys ratios of 124, 120, 126, 109, and 108%, respectively. Although the variability in the ratios reported by these researchers tended to be lower, the results observed in experiment 3 were ratios between 100 and 113%.

Decreased weight gain and poorer feed conversion ratio were observed when a 121% Leu-to-Lys ratio was supplied. This may be due to the antagonist effect of excessive Leu on valine and isoleucine metabolism. Working with rats, May et al. (1991) found that excess Leu impaired the performance of rats fed low-protein diets.

In conclusion, the recommended digestible Phe + Tyr-to-Lys, digestible His-to-Lys, and digestible Leu-to-Lys ratios to meet the requirements for the main production parameters of broilers during the starter phase (8 to 17 d of age) are 104% (Table 5).
